# 73 Fibroblasts Derived from Scar Lose Fibrotic Phenotype in Culture: Are We Using the Right Model?

**DOI:** 10.1093/jbcr/irad045.047

**Published:** 2023-05-15

**Authors:** Bonnie Carney, Lauren Moffatt, Taryn Travis, Jeffrey Shupp

**Affiliations:** Firefighters' Burn and Surgical Research Laboratory, Medstar Health Research Institute, Georgetown University Medical Center, Washington, DC; Medstar Health, Washington, DC; MedStar Washington Hospital Center, Washington, DC; Firefighters' Burn and Surgical Research Laboratory, MedStar Health Research Institute, Georgetown Univeristy School of Medicine, Washington, DC; Firefighters' Burn and Surgical Research Laboratory, Medstar Health Research Institute, Georgetown University Medical Center, Washington, DC; Medstar Health, Washington, DC; MedStar Washington Hospital Center, Washington, DC; Firefighters' Burn and Surgical Research Laboratory, MedStar Health Research Institute, Georgetown Univeristy School of Medicine, Washington, DC; Firefighters' Burn and Surgical Research Laboratory, Medstar Health Research Institute, Georgetown University Medical Center, Washington, DC; Medstar Health, Washington, DC; MedStar Washington Hospital Center, Washington, DC; Firefighters' Burn and Surgical Research Laboratory, MedStar Health Research Institute, Georgetown Univeristy School of Medicine, Washington, DC; Firefighters' Burn and Surgical Research Laboratory, Medstar Health Research Institute, Georgetown University Medical Center, Washington, DC; Medstar Health, Washington, DC; MedStar Washington Hospital Center, Washington, DC; Firefighters' Burn and Surgical Research Laboratory, MedStar Health Research Institute, Georgetown Univeristy School of Medicine, Washington, DC

## Abstract

**Introduction:**

Hypertrophic scar (HTS) after burn injury is difficult to treat and leads to lasting morbidity. As such, HTS formation and treatment strategies are essential to study in the laboratory. Animal models of HTS exist, but are limited by high cost and the small numbers of scars that can be created for study. As an alternative, the isolation of HTS cells for study in the laboratory is a common practice. In this study, we examine the maintenance of fibrotic fibroblast phenotype in vitro to determine whether cells retain characteristics of HTS. We hypothesized that these features may not persist in culture.

**Methods:**

HTSs were formed in Duroc pigs (n=4 scars). On day 105 post-injury, tissue biopsies were obtained from HTS and normal skin (NS). Biopsies were either: saved immediately by flash freezing; stored in dispase solution for overnight incubation to remove the epidermis and then frozen; or incubated in 1 mg/mL collagenase for 6 hours at 37^o^C to obtain single cell suspensions. These single cells were harvested with Trizol reagent immediately prior to seeding, or were seeded, grown, and harvested 24 hours later. Cells were seeded in DMEM/F12 media with 10% FBS and 1% pen/strep. In a subset of cells, 18% macromolecular crowding (MMC) was achieved through addition of 70/400kDa ficoll to media. RNA was isolated from tissues and cells. qRT-PCR was performed for collagen 1a1 (COL1A1) and galectin 1 (LGALS1). HTS was normalized to NS.

**Results:**

HTSs had increased stiffness, trans-epidermal water loss, and dyschromia compared to NS (p< 0.05). COL1A1 expression in biopsies harvested from *in vivo* HTS was 18.96-fold greater compared to NS. After 24 hours at 4^o^C, this level dropped to 6.28-fold. Cells isolated from immediate processing lost all differential COL1A1 expression in HTS vs. NS (1.75-fold). After 24 hours in culture, fibroblasts down-regulated COL1A1 expression in HTS vs. NS (-2.39-fold). The addition of MMC media to mimic excessive extracellular matrix did not change COL1A1 expression (-2.65-fold). Similarly, for LGALS1, *in vivo* biopsies had high expression at 3.52-fold in HTS vs. NS and each group thereafter had reduced expression (6.68, 1.09, -1.19, -2.69).

**Conclusions:**

When fibroblasts are isolated from HTS and digested for use in cell culture experiments, their phenotype changes immediately upon isolation of the cells even prior to seeding. This suggests that additional steps are needed for HTS fibroblast cell isolation to retain HTS cell expression.

**Applicability of Research to Practice:**

Current methods for isolating scar single cells are likely not nuanced enough to capture the HTS phenotype. This could contribute to the lack of translation of findings from the *in vitro* environment to clinical scar treatments. Additional research should focus on optimizing methods to culture HTS fibroblasts that are phenotypically similar to *in vivo* scars in order to study scar interventions. These findings also have implications for single cell sequencing experiments.

